# Application of digital design and 3D printing technology in repairing mandibular defects with fibula musculocutaneous flap

**DOI:** 10.1097/MD.0000000000043883

**Published:** 2025-08-22

**Authors:** Chao Wang, Yue Li, Yan Zhang, Wei Chen, Ying bin Yan, Jun Zhang

**Affiliations:** a Department of Oral and Maxillofacial Surgery, Tianjin Stomatological Hospital, School of Medicine, Nankai University, Tianjin, China; b Tianjin Key Laboratory of Oral and Maxillofacial Function Reconstruction, Tianjin, China.

**Keywords:** 3D printing technology, digital design, fibular musculocutaneous flap, mandibular defect

## Abstract

**Background:**

Mandibular tumors are common in oral and maxillofacial surgery. Following tumor resection, significant bone tissue defects often occur, severely compromising both facial appearance and function. Currently, fibular myocutaneous flaps are widely employed by head and neck surgeons to repair various types of mandibular defects. However, accurately restoring the mandibular contour remains a major challenge. To achieve optimal clinical outcomes, we applied digital design and 3D printing technology in conjunction with fibula musculocutaneous flaps for mandibular defect reconstruction. In this study, we present our approach and share the experience gained.

**Methods:**

A retrospective review was conducted on 16 patients who underwent mandibular reconstruction between June 2021 and May 2024. All patients had mandibular bone defects following oral tumor resection and were treated using vascularized fibula myocutaneous flaps guided by digital design and 3D printing technology.

**Results:**

Flap survival was achieved in 15 patients (93.7%), with 1 case (6.3%) of flap necrosis. During a follow-up period of 6 to 30 months, 1 patient (6.3%) experienced local tumor recurrence. All followed-up patients reported satisfactory outcomes in terms of mouth opening, masticatory function, and facial aesthetics.

**Conclusion:**

The integration of digital design and 3D printing technology in mandibular reconstruction using fibula musculocutaneous flaps facilitates accurate anatomical restoration and improves facial symmetry, contributing to favorable clinical outcomes.

Tissue defect after mandibular tumor operation significantly impair patients’ quality of life. Effective reconstruction is essential to restore mandibular integrity, maintain oral aperture and facial symmetry, and provide adequate bone support for future implant-based dental rehabilitation. From June 2021 to May 2024, 16 patients admitted to the maxillofacial Surgery Department of Tianjin Stomatology Hospital underwent mandibular reconstruction using vascularized fibula musculocutaneous flap assisted by digital design and 3D printing technology. Satisfactory clinical outcomes were achieved.

## 1. Introduction

A total of 16 patients were enrolled in this study, comprising 11 males and 5 females, aged between 19 and 67 years (mean age: 41 years). Among them, 7 patients were diagnosed with gingival cancer (according to TNM stage, 4 patients with T2N0M0 and 3 patients with T3N0M0), and 9 cases were diagnosed with ameloblastoma. The site of the disease involved unilateral mandibular body, the middle of mandibular chin, the junction of the mandibular body and ascending ramus, and the ascending branch of mandible involved condylar process. According to the Urken classification of mandible defects,^[1]^ there were 3 cases of condyle and ramus and body defects, 4 cases of ramus and body defects, 1 case of ramus and body and symphyseal defects, 1 case of body and symphyseal and symphyseal and body defects, 2 cases of symphyseal and body and ramus defects, 3 cases of body and ramus defects and 2 cases of body and ramus and condyle defects (Table 1). Cone beam computed tomography (CBCT) examination of mandible and spiral CT examination of lower extremity in donor area were performed in all patients. CT data of each patient were collected and imported into Mimics 20.0 software to perform digital virtual osteotomy design.

**Table 1:** Patient data.

Case number	Age (yr)	Sex	Tumor	Stage	Defect area	PML (h/a) (cm)	PMABH (h/a) (cm)
1	21	M	OA	–	RB	10.9/10.7	6.4/6.1
2	45	F	SCC	T2N0M0	SBR	11.6/12.1	6.5/6.2
3	35	F	OA	–	BR	9.3/9.6	5.4/5.7
4	19	M	OA	–	BRC	10.6/10.2	6.9/7.2
5	55	M	SCC	T2N0M0	BSSB	12.3/11.6	7.1/6.8
6	51	M	OA	–	SBR	10.1/10.4	6.8/6.5
7	42	M	SCC	T2N0M0	RB	10.6/9.9	7.4/7.1
8	26	M	OA	–	RB	10.4/10.7	6.3/6.1
9	38	F	OA	–	RBS	11.3/10.5	6.5/6.8
10	52	M	SCC	T3N0M0	CRB	11.4/11.7	6.6/6.3
11	33	F	OA	–	BR	10.3/9.8	7.1/6.7
12	49	M	SCC	T2N0M0	BRC	10.1/10.5	6.8/7.2
13	28	M	OA	–	RB	10.9/11.3	6.5/6.9
14	34	M	OA	–	BR	10.8/10.4	7.2/6.7
15	61	F	SCC	T3N0M0	CRB	10.2/9.7	6.7/6.3

BR = body and ramus, BRC = body and ramus and condyle, BSSB = body and symphyseal and symphyseal and body, CRB = condyle and ramus and body, OA = odontoameloblastoma, PML (h/a) = postoperative mandibular length (healthy side/affected side), PMABH (h/a) = postoperative mandibular ascending branch height (healthy side/affected side), RB = ramus and body, RBS = ramus and body and symphyseal, SBR = symphyseal and body and ramus, SCC = squamous cell carcinoma of the gingiva.

**Figure 1.**
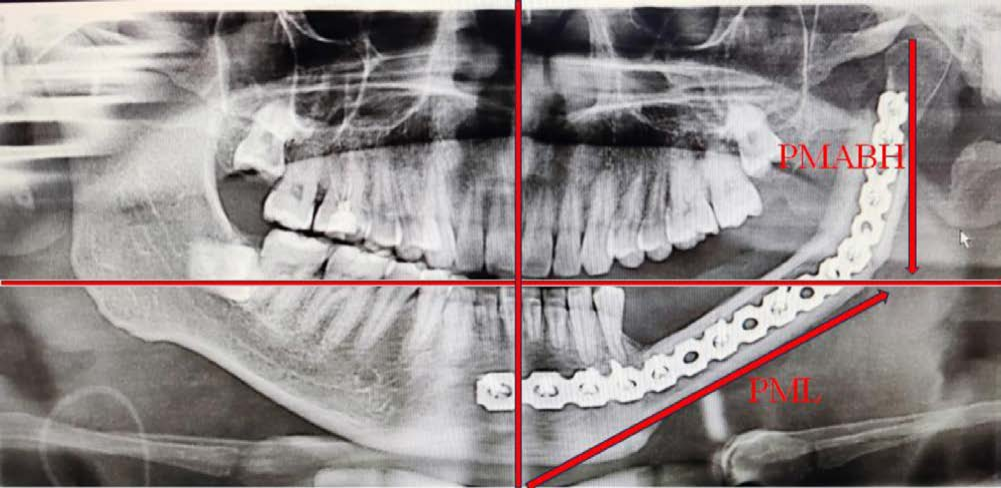
Schematic diagram of measuring method of mandibular body length and ascending branch height.

The osteotomy guides, the shape guides, and the positioning guides were made to assist the surgical procedure. The mandible was reconstructed with vascularized fibula musculocutaneous flap following mandibular tumor resection. Postoperative evaluation of mandibular body length and ramus height was conducted using X-ray imaging (Fig. 1). All patients involved in the study have full right to know and were fully informed. This study was approved by the Ethics Committee of Tianjin Stomatological Hospital (Approval Document Number: 2023-SYDWLL-000282).

## 2. Preoperative virtual operation design and guide plate making

Preoperative color Doppler ultrasonography of the lower extremity vessels was performed to exclude the presence of a common trunk between the posterior tibial artery and the peroneal artery. Spiral CT examination was performed on the lower limb and CBCT examination was performed on the maxillofacial region. The data of spiral CT and CBCT were saved as DICOM format and imported into Mimics 20.0 software. A surgical margin of 1.5–2 cm was maintained around malignant tumors, while a 5 mm margin was used for resection of ameloblastomas. Based on the pathological extent of the mandibular lesion, virtual osteotomy was simulated on the 3D model. The required length and segmentation of the fibula graft were determined according to the size and location of the defect. For unilateral defects, reconstruction typically involved 2 or 3 fibula segments. In some cases, segmental folding or supplementary free bone grafting was employed to increase the vertical height of the reconstructed mandible, thereby providing an optimal foundation for future implant-based rehabilitation. To ensure the stability of the ankle and knee joints, the lower end of the fibula was positioned 8 cm proximal to the center of the ankle and the upper end was placed 6 cm distal to the small head of the fibula. The external side of the fibula is designed as the external side of the mandible. According to the confirmed osteotomy scheme, the osteotomy guide, the shaping guide and the positioning guide were designed. A 3D model of the mandible was then reconstructed, and the guides were fabricated using a 3D printer (Ender 5s).

## 3. Surgical methods

### 3.1. Primary lesion excision

After general anesthesia, the patient was catheterized and covered with towel. For patients with gingival cancer, cervical lymph node dissection was performed first, followed by segmental osteotomy of the jaw bone. A lower lip-splitting incision combined with elevation of the cheek flap was employed to achieve full exposure of the lesion and the mandibular region. Preoperatively designed anterior and posterior osteotomy guides were positioned and secured with screws. Upon completion of the osteotomy, the guide plates were removed and the positioning guide was installed to assist with flap reconstruction.

### 3.2. Preparation of fibula musculocutaneous flap

Simultaneously with resection of the primary lesion, 2 sets of operations were generally performed. The conventional Henry approach was applied. The skin, subcutaneous fat and fascia lata were sequentially incised to expose the peroneus longus, peroneus brevis, and posterolateral intermuscular septum. The posterior lateral intermuscular septa of the calf was bluntly dissected from front to back on the superficial surface of the long and short peroneal muscle and the deep fascia lata, exposing the perforating branch of the peroneal artery. The lateral border of the calf was palpated as an anatomical marker to detach the origins of the peroneus longus and brevis muscles. These were removed from the lateral side of the fibula to the anterior edge of the fibula. The attachment of the extensor long toe and great extensor long muscle were separated along the anterior edge of the fibula to expose the anterior compartment of the calf, with careful preservation of the anterior tibial vascular bundle and the deep peroneal nerve. The perforating branch of peroneal artery was used as the center to design the island. The posterior margin of the skin island was incised, and the flap was elevated from the deep fascia to expose the posterior compartment and identify the posterior tibial vessels and tibial nerve. The penetration point of the peroneal artery on the surface of the flexor hallucis longus was located. The flexor hallucis longus was incised with care to protect the perforating branch, which was then dissected proximally to its origin from the peroneal artery. The fibula was transected with a wire saw: the lower end 8 cm above the lateral malleolus, and the upper end 6 cm below the fibular head. The common peroneal nerve, posterior tibial vascular bundle, and tibial nerve were carefully preserved. The interosseous membrane was incised in the anterior compartment of the lower leg to expose the posterior tibialis muscle. At the lower end of the fibula, the distal end of the peroneal vessels was identified, ligated, and transected. The fibula was retracted laterally while the flexor hallucis longus and posterior tibialis muscles were separated along the vascular bundle from distal to proximal, preserving a 5 mm muscle cuff to protect the bundle. Finally, the vascular pedicle was dissected to its origin from the posterior tibial artery, and a vessel loop was prepared for eventual division.

### 3.3. Mandibular shaping and reconstruction

The vascular pedicle was cut and ligated, and the fibular myocutaneous flap was transferred to the recipient site. The pedicle was carefully separated from the bone at the planned osteotomy location. A fibular osteotomy guide plate was then positioned on the fibula according to the preoperative design. The excess, nonfunctional bone segment was removed, and the remaining bone was aligned with the positioning guide plate for shaping. The bone segment was adjusted and polished to correct any osteotomy inaccuracies and to ensure a precise fit with the shaping guide. Finally, the bone segment was provisionally fixed in place in preparation for vascular anastomosis.

### 3.4. Vascular anastomosis

Following the shaping of the fibular segment, vascular anastomosis was performed, beginning with arterial anastomosis. Typically, an end-to-end anastomosis was carried out between the peroneal artery and either the facial artery or the superior thyroid artery. After assessing arterial flow and ensuring satisfactory blood return, venous anastomosis was performed. Depending on venous return, the facial vein, a branch of the internal jugular vein, or the external jugular vein was selected for end-to-end anastomosis with the peroneal vein. Once adequate vascular perfusion of the flap was confirmed, the bone segments were securely fixed using internal fixation. Complete hemostasis was achieved, negative pressure drainage was placed, and the surgical wound was closed. Postoperative care of the flap was conducted according to standard monitoring and nursing protocols.

## 4. Result

Among the 16 patients, flap survival was achieved in 15 cases, while 1 patient with gingival carcinoma experienced flap necrosis. Postoperatively, facial contours were generally symmetrical in the 15 surviving cases, and no discomfort in the bilateral temporomandibular joint area was reported during the 6 to 30-month follow-up period. One patient experienced local tumor recurrence. All followed-up patients reported satisfaction with mouth opening, masticatory function, and facial aesthetics.

## 5. Typical case

A 35-year-old female patient was admitted to hospital with the diagnosis of “left mandibular ameloblastoma.”

Clinical manifestations: The patient’s exhibited mild swelling along the left facial profile. Mouth opening was normal, with the interincisal distance of approximately 3.7 cm. Occlusal alignment was normal, and the mass protruded in the posterior region of the left mandibular molar. CT showed the destruction of the left mandibular body and the anterior edge of the ascending ramus.

**Figure 2.**
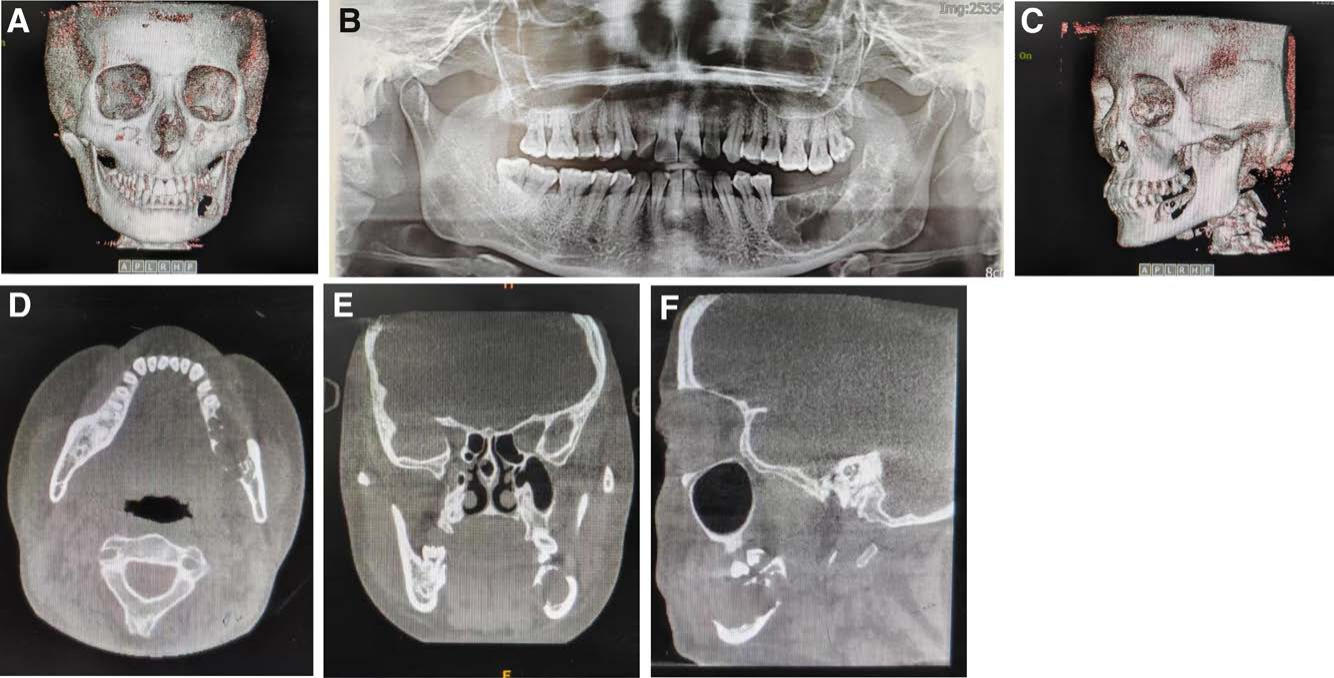
The surface tomography and CT image manifestations of patient. (A) Preoperative frontal image of the jawbone. (B) The preoperative surface tomographic images of patient. (C) Preoperative lateral image of the jawbone. (D) Horizontal scan manifestation. (E) Coronal scan manifestation. (F) Sagittal scan manifestation.

**Figure 3.**
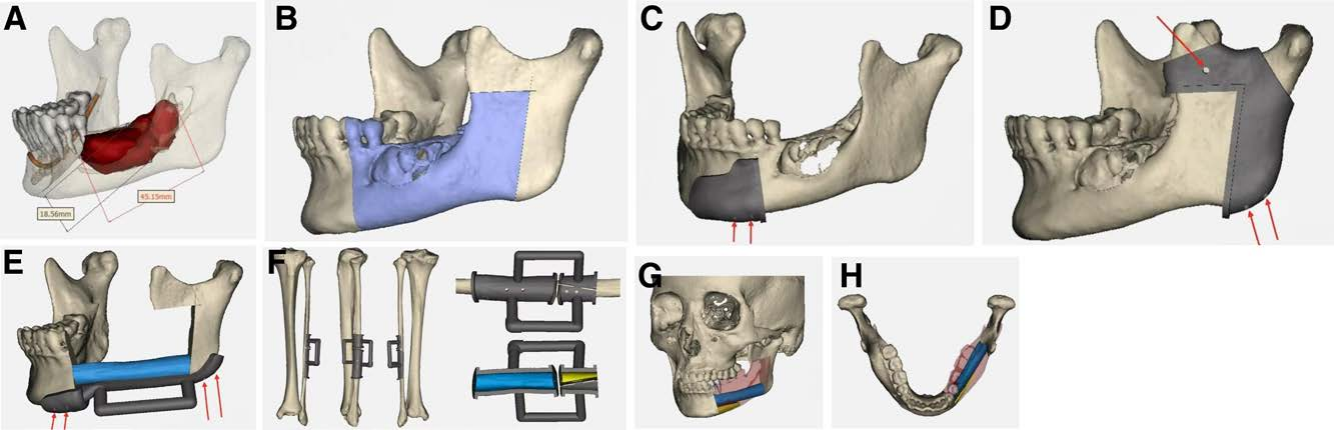
Digital design of osteotomy and repair methods. (A) Determine the range of the lesion. (B) Simulate the range of bone cutting. (C) Design the anterior bone cutting guide. (D) Design the rear bone cutting guide. (E) Design the positioning guide (F) Design the fibula osteotomy guide. (G) Restore the mandibular contour in lateral view (H) Restore the mandibular contour in superior view (The red arrow shows the positioning holes).

**Figure 4.**
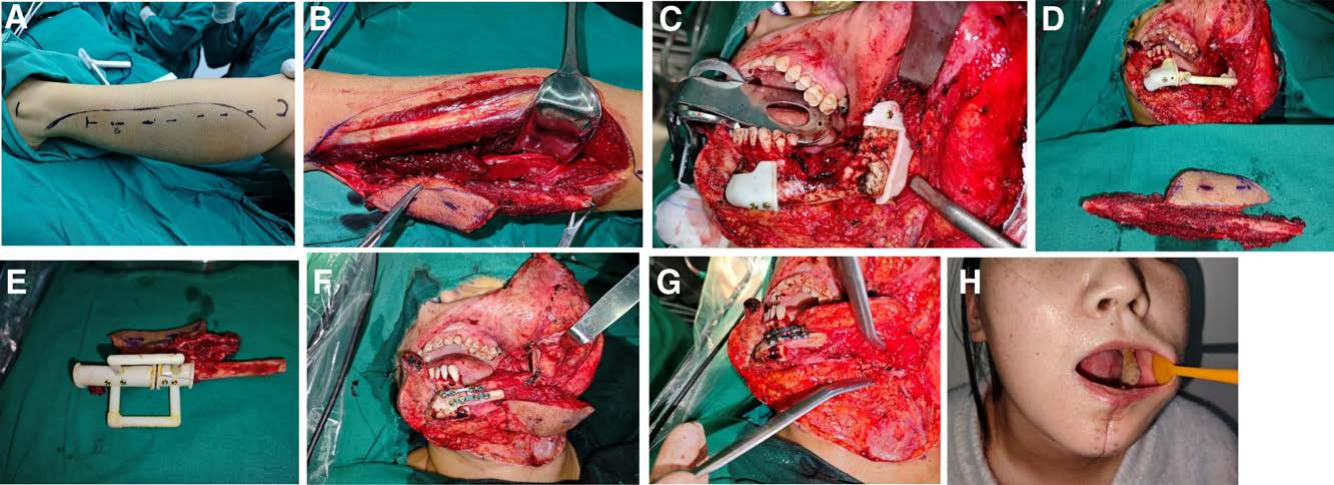
Application of 3D printing guide to assist osteotomy and reconstruction of mandibular defect. (A) Fibula resection approach. (B) Fibula myocutaneous flap was prepared. (C) The lesion was fully exposed and the osteotomy guide was placed. (D) Fibular myocutaneous flap with severed pedicle and positioning guide plate (E) Osteotomy guide plate of fibula. (F) Finished shaping and rigid internal fixation (G) Complete vascular anastomosis. (H) Oral endoscopy 1 month after surgery.

**Figure 5.**
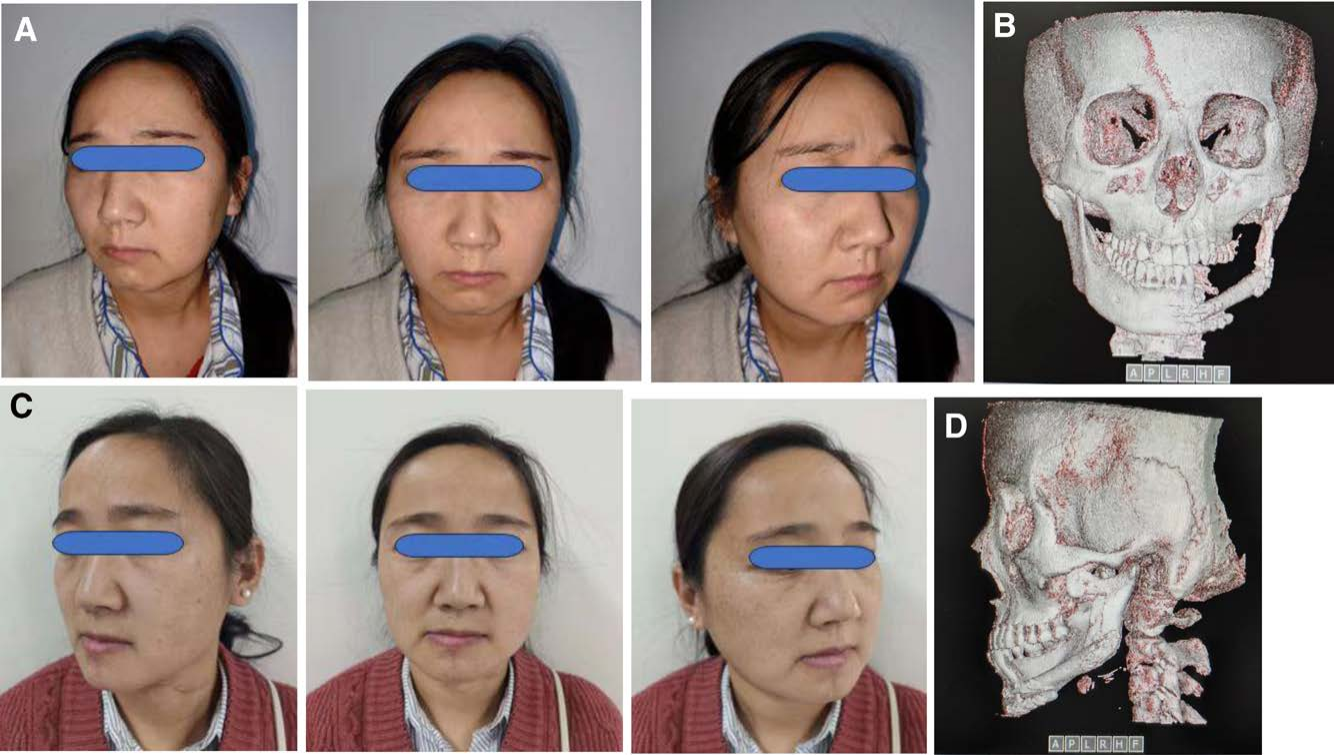
Comparison of preoperative and postoperative images of patient. (A) Preoperative image of patient. (B) Postoperative frontal image of the jawbone. (C) Postoperative image of patient. (D) Postoperative lateral image of the jawbone.

**Figure 6.**
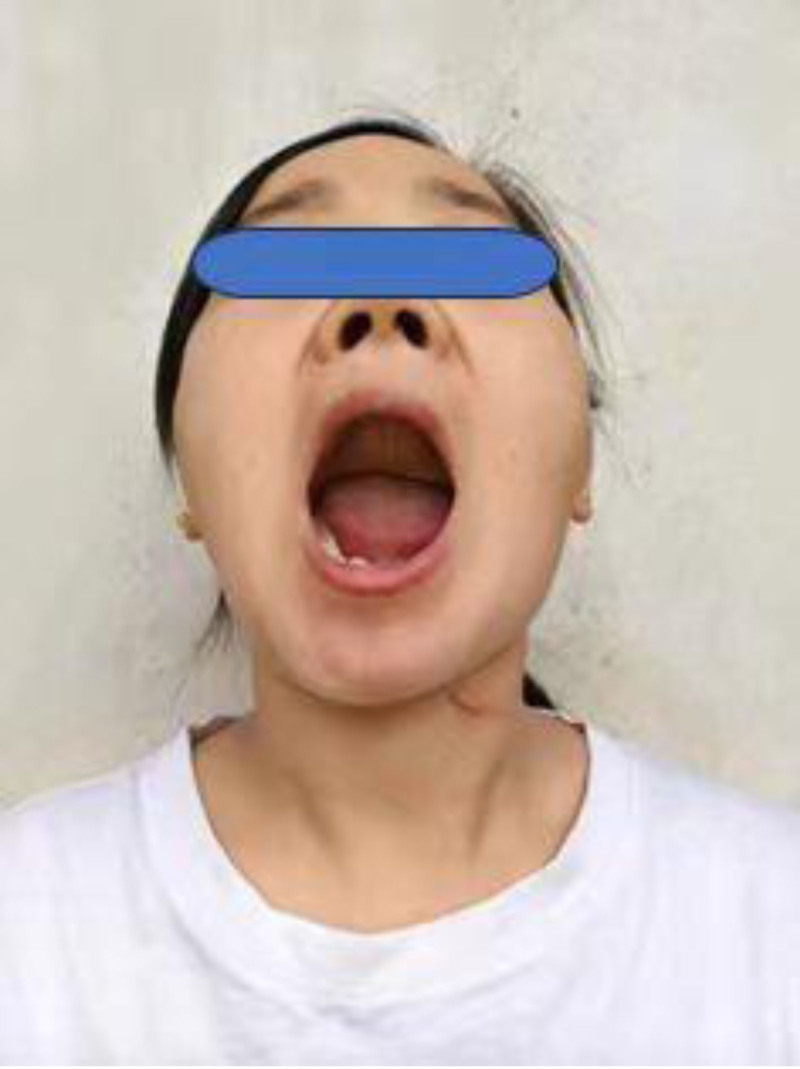
The opening degree of the patient 6 months after the operation. The opening is approximately 38 mm.

Treatment methods: CT data of the left lower limb and jaw bone were collected and imported into virtual planning software for digital design (Figs. 2 and 3). According to the design scheme, 3D printing technology is applied to fabricate the osteotomy guide, shaping guide and positioning guide. Osteotomy and reconstruction of mandibular defect were performed according to these guide plates (Fig. 4). Postoperatively, the patient was satisfied with the restoration of her facial contours (Figs. 5 and 6).

## 6. Discussion

Mandibular defects can disrupt mandibular continuity and result in significant functional impairments, seriously affecting a patient’s appearance, mastication, speech, and swallowing. The fibula, a non-weight-bearing bone of the lower limb, is an ideal donor site for mandibular reconstruction. As long as at least 7 cm of the distal fibula is preserved, ankle joint stability can be maintained. In addition, the fibula provides a sufficiently long bone segment and has a stable vascular supply, making it particularly well-suited for mandibular repair and reconstruction. As such, the vascularized free fibular myocutaneous flap has become the standard approach for mandibular defect reconstruction.^[2,3]^ Computer-assisted surgery offers a powerful platform for virtual preoperative planning, allowing surgeons to design and simulate precise surgical procedures more efficiently.^[4,5]^ With the integration of digital technologies – including computer-aided design, computer-aided manufacturing, reverse engineering, 3D printing, rapid prototyping, and surgical navigation – it is now possible to achieve personalized and accurate reconstruction of mandibular defects. These advancements contribute to the restoration of mandibular continuity, facial contour, and essential oral functions.^[6–10]^ This study primarily investigated the use of a fibular myocutaneous flap, combined with a computer-designed and 3D-printed surgical guide, to achieve accurate mandibular defect repair and reconstruction. Among the 16 patients treated, flap necrosis occurred in 1 case due to venous crisis; the flap was removed, and the wound was closed locally. In the remaining 15 patients, all flaps survived. Of these, 9 patients retained the native condyle on the affected side, which was appropriately repositioned in the articular fossa postoperatively. In 6 patients, the mandibular condyle was reconstructed using the fibular flap. Postoperative evaluation was conducted using radiographic imaging. A 2-sample *t* test was used to analyze differences in mandibular body length and ramus height between the affected and unaffected sides. The results indicated no statistically significant differences, suggesting that bilateral mandibular dimensions were well preserved. These findings support the conclusion that the proposed technique enables precise anatomical reconstruction, restoring both structure and function of the mandible.

Maintaining the physiological position of the condyle after mandibular continuity is disrupted remains a significant challenge – particularly in cases involving large mandibular defects. Yuan et al addressed this by designing a surgical guide based on the osteotomy surface, which was fixed at the superior margin of the remaining bone segment after osteotomy.^[11]^ However, if the osteotomy deviated intraoperatively, the condylar position could become misaligned. To overcome this, Numajiri et al developed a 2-in-1 guide to assist in condylar positioning.^[12,13]^ Although effective in localizing the condyle, the additional fixation device could interfere with subsequent surgical steps. In our approach, osteotomy was performed under the guidance of a custom-designed osteotomy guide. Following this, a condylar positioning guide was used to align and fix the remaining bone segments according to the preoperative design. This ensured that the postoperative condylar position closely matched the natural anatomical position observed in the preoperative CT scan. The same condylar positioning guide was also used in the recipient site as a shaping guide. After osteotomy of the fibular bone segment in the donor area, the shaped segment was transferred to the recipient site and fine-tuned under guidance. This allowed for precise reconstruction of the mandibular defect using the positioning guide. Once all free bone segments were securely fixed, the condylar positioning guide was removed, leaving the condyle accurately restored to its physiological position. In cases where the condyle was not preserved, a separate positioning guide was designed for the recipient site. Since minor discrepancies may arise at each osteotomy interface, the guide was instrumental in compensating for cumulative error. This ensured accurate restoration of mandibular width and height, ultimately achieving an optimal postoperative submandibular contour.

After resection of a mandibular tumor, the residual bone segment often becomes unstable. In traditional treatment, shaping the fibula relies heavily on the surgeon’s experience. However, the fibula is a dense bone that is difficult to contour intraoperatively, often requiring a significant amount of time and effort.^[14,15]^ Computer-aided digital virtual surgical planning allows for precise bone cutting, shaping, and restoration of mandibular anatomy. Translating this virtual osteotomy plan accurately into clinical practice is critical. The use of 3D-printed surgical guide plates offers an effective solution to this challenge. Based on our clinical experience, we summarize the following key considerations for the effective application of guide plates: Material strength: Guide plates are typically fabricated from acrylonitrile butadiene styrene plastic. The material must possess sufficient mechanical strength; otherwise, the guide may fracture during screw fixation or osteotomy. Base fit and positioning: The base of the guide plate should fit the bone surface as closely as possible. A closer fit ensures higher surgical precision. It is advisable to design the base to rest on areas with natural bony prominences for better stability. If such landmarks are absent at the osteotomy site, the base can be extended to reach a more secure location, improving fixation, and positioning accuracy. Saw groove design: The width of the saw groove must be carefully optimized. A groove that is too wide increases osteotomy error, while 1 that is too narrow risks jamming during cutting. We recommend a clearance of 1.5 mm for the saw groove to facilitate smooth bone cutting and minimize deviation.

The application of digital design and 3D printing technology in fibular reconstruction of mandibular defects is highly promising and worthy of broader clinical adoption. The use of mirror-reference models allows for precise restoration of mandibular body curvature, mandibular angle, and body height. In cases involving condyle preservation, the position of the condyle can be accurately determined, enabling accurate anatomical reconstruction of the mandible and thereby improving the restoration of facial contour and oral function. Moreover, digital design and 3D printing technology can significantly streamline the surgical procedure. Preoperative bending of the titanium fixation plate can be completed using a patient-specific 3D model. Bone cutting can be performed precisely according to the mandibular osteotomy guide, and fibular shaping can be accurately achieved using the shaping and positioning guides. This level of precision not only enhances surgical accuracy but also greatly reduces operative time. Shortening the duration of surgery has additional benefits: it reduces surgeon fatigue and minimizes the likelihood of human error, thereby lowering the risk of postoperative complications.^[16]^

In conclusion, the accurate reconstruction of mandibular defects using a fibular myocutaneous flap remains a complex and demanding task in oral and maxillofacial surgery. The integration of digital design and 3D printing technology into the reconstruction process enables not only precise defect repair and preservation of oral function but also a reduction in operative time and postoperative complications. These advantages contribute to improved surgical outcomes and patient satisfaction. Therefore, the use of vascularized fibular myocutaneous flaps, guided by digital planning and 3D printing technology, represents a highly effective and clinically valuable approach that merits broader clinical application.

## Author contributions

**Conceptualization:** Chao Wang.

**Data curation:** Chao Wang, Yue Li, Yan Zhang, Wei Chen.

**Formal analysis:** Chao Wang, Yue Li, Ying bin Yan.

**Investigation:** Yue Li, Yan Zhang.

**Methodology:** Chao Wang, Yan Zhang.

**Resources:** Ying bin Yan.

**Software:** Chao Wang.

**Writing – original draft:** Chao Wang.

**Writing – review & editing:** Jun Zhang.
